# The Effect of Surgical Resection on Cancer-Specific Mortality in Pelvic Soft Tissue Sarcoma According to Histologic Subtype and Stage

**DOI:** 10.3390/jcm13195787

**Published:** 2024-09-28

**Authors:** Mattia Luca Piccinelli, Andrea Baudo, Stefano Tappero, Cristina Cano Garcia, Francesco Barletta, Reha-Baris Incesu, Simone Morra, Lukas Scheipner, Zhe Tian, Stefano Luzzago, Francesco Alessandro Mistretta, Matteo Ferro, Fred Saad, Shahrokh F. Shariat, Sascha Ahyai, Nicola Longo, Derya Tilki, Alberto Briganti, Felix K. H. Chun, Carlo Terrone, Luca Carmignani, Ottavio de Cobelli, Gennaro Musi, Pierre I. Karakiewicz

**Affiliations:** 1 Cancer Prognostics and Health Outcomes Unit, Division of Urology, University of Montréal Health Center, Montreal, QC H2X 3E4, Canada; 2 Department of Urology, IEO European Institute of Oncology, IRCCS, Via Ripamonti 435, 20141 Milan, Italy; 3 School of Urology, Università degli Studi di Milano, 20122 Milan, Italy; 4 Department of Urology, IRCCS Policlinico San Donato, 20097 Milan, Italy; 5 Department of Urology, IRCCS Policlinico San Martino, 16132 Genova, Italy; 6 Department of Surgical and Diagnostic Integrated Sciences (DISC), University of Genova, 16132 Genova, Italy; 7 Department of Urology, University Hospital Frankfurt, Goethe University Frankfurt am Main, 60590 Frankfurt am Main, Germany; 8 Division of Experimental Oncology, Unit of Urology, URI, Urological Research Institute, IRCCS San Raffaele Scientific Institute, 20132 Milan, Italy; 9 Martini-Klinik Prostate Cancer Center, University Hospital Hamburg-Eppendorf, 20251 Hamburg, Germany; 10 Department of Neurosciences, Science of Reproduction and Odontostomatology, University of Naples Federico II, 80131 Naples, Italy; 11 Department of Urology, Medical University of Graz, 8010 Graz, Austria; 12 Department of Oncology and Haemato-Oncology, Università degli Studi di Milano, 20122 Milan, Italy; 13 Department of Urology, Comprehensive Cancer Center, Medical University of Vienna, 1090 Vienna, Austria; 14 Department of Urology, Weill Cornell Medical College, New York, NY 10065, USA; 15 Department of Urology, University of Texas Southwestern Medical Center, Dallas, TX 75390, USA; 16 Hourani Center of Applied Scientific Research, Al-Ahliyya Amman University, Amman 19328, Jordan; 17 Department of Urology, University Hospital Hamburg-Eppendorf, 20246 Hamburg, Germany; 18 Department of Urology, Koc University Hospital, 34010 Istanbul, Turkey; 19 Department of Urology, IRCCS Ospedale Galeazzi—Sant’Ambrogio, 20157 Milan, Italy

**Keywords:** soft tissue sarcoma, pelvis, cancer-specific mortality

## Abstract

**Background/Objectives**: The impact of surgical resection versus non-resection on cancer-specific mortality (CSM) in soft tissue pelvic sarcoma remains largely unclear, particularly when considering histologic subtypes such as liposarcoma, leiomyosarcoma, and sarcoma NOS. The objective of the present study was to first report data regarding the association between surgical resection status and CSM in soft tissue pelvic sarcoma. **Methods**: Using data from the Surveillance, Epidemiology, and End Results (SEER) database from 2000 to 2019, we identified 2491 patients diagnosed with pelvic soft tissue sarcoma. Cumulative incidence plots were used to illustrate CSM and other-cause mortality rates based on the histologic subtype and surgical resection status. Competing risk regression models were employed to assess whether surgical resection was an independent predictor of CSM in both non-metastatic and metastatic patients. **Results**: Among the 2491 patients with soft tissue pelvic sarcoma, liposarcoma was the most common subtype (41%), followed by leiomyosarcoma (39%) and sarcoma NOS (20%). Surgical resection rates were 92% for liposarcoma, 91% for leiomyosarcoma, and 58% for sarcoma NOS in non-metastatic patients, while for metastatic patients, the rates were 55%, 49%, and 23%, respectively. In non-metastatic patients who underwent surgical resection, five-year CSM rates by histologic subtype were 10% for liposarcoma, 32% for leiomyosarcoma, and 27% for sarcoma NOS. The multivariable competing risk regression analysis showed that surgical resection provided a protective effect across all histologic subtypes in non-metastatic patients (liposarcoma HR: 0.2, leiomyosarcoma HR: 0.5, sarcoma NOS HR: 0.4). In metastatic patients, surgical resection had a protective effect for those with leiomyosarcoma (HR: 0.6) but not for those with sarcoma NOS. An analysis for metastatic liposarcoma was not possible due to insufficient data. **Conclusions:** In non-metastatic soft tissue pelvic sarcoma, surgical resection may be linked to a reduction in CSM. However, in metastatic patients, this protective effect appears to be limited primarily to those with leiomyosarcoma.

## 1. Introduction

Soft tissue pelvic sarcoma is an uncommon mesenchymal malignancy, comprising roughly 5% of all soft tissue sarcomas, with an estimated incidence of less than 0.1 per 100,000 cases per year [1,2]. While most cases occur sporadically, up to 3% are linked to genetic syndromes such as Li-Fraumeni, retinoblastoma, neurofibromatosis type 1 and familial adenomatous polyposis [3]. Additionally, chronic lymphedema and prior radiation therapy for gynecologic, rectal, or prostate cancers have been identified as risk factors [4]. Despite being a different entity, soft tissue pelvic sarcomas are frequently included in broader studies that encompass sarcomas from different anatomical locations. Although there are similarities between soft tissue pelvic sarcoma and retroperitoneal sarcoma, notable differences exist in histologic subtypes, surgical management, recurrence patterns, and survival outcomes [5,6,7].

The management of soft tissue pelvic sarcoma is complex due to the anatomical constraints of the pelvis, which lacks the distinct fascial compartments present in the extremities. This, combined with often delayed diagnoses, typically results in larger tumor sizes at presentation, making complete surgical resection challenging or sometimes impossible. Symptoms commonly arise from the tumor’s mass effect or its invasion into adjacent pelvic organs or vessels [8]. The proximity of these tumors to critical structures within the confined pelvic space often necessitates complex surgical approaches, including multiorgan resections and occasionally vascular resections with or without reconstruction. The mainstay of treatment for localized soft tissue pelvic sarcoma is radical surgical resection, which remains the only curative option [2,9,10]. However, in cases of metastatic sarcoma, the role of surgery is primarily palliative, aimed at managing local symptoms, and its impact on overall cancer control remains uncertain [11,12,13].

Soft tissue pelvic sarcomas most commonly present as liposarcoma and leiomyosarcoma, which together account for 30–35% of all soft tissue sarcomas [2,14]. Significant variations in local recurrence (20–41%) and metastatic progression (20–50%) rates have been observed at five years, depending on the histologic subtype, particularly in retroperitoneal sarcomas. Despite these differences, cancer control outcomes in pelvic sarcomas based on the histologic subtype have only been explored in small, retrospective studies, underscoring the need for larger, more comprehensive studies [2,15].

Given the unique challenges posed by soft tissue pelvic sarcomas, there is a critical need for a better understanding of the relationship between surgical resection and cancer-specific mortality (CSM) in both non-metastatic and metastatic settings. Currently, there are no robust data on the association between resection status and CSM for either non-metastatic or metastatic soft tissue pelvic sarcomas. Survival data for soft tissue pelvic sarcomas tend to be inconsistent and largely derived from small, historical cohorts [16,17]. This lack of evidence limits the ability of clinicians to make informed decisions regarding the potential benefits of surgical intervention in these patients.

In this study, we sought to address these knowledge gaps using data from the Surveillance, Epidemiology, and End Results (SEER) database spanning from 2000 to 2019. We aimed to evaluate whether surgical resection is associated with lower CSM in non-metastatic patients and whether this association varies according to the three most common histologic subtypes, namely liposarcoma, leiomyosarcoma, and sarcoma not otherwise specified (NOS). We hypothesized that surgical resection would be linked to a reduction in CSM across all histologic subtypes in non-metastatic patients, but that this association might be differentially affected by histology in the metastatic setting. Specifically, we postulated that even among metastatic patients, surgical resection could be associated with more favorable CSM outcomes.

This investigation represents one of the most extensive analyses to date on soft tissue pelvic sarcomas, utilizing a robust national database to explore the impact of surgical resection across diverse clinical scenarios. Our findings could have significant implications for the clinical management of soft tissue pelvic sarcomas, providing a more nuanced understanding of the role of surgery in both localized and metastatic disease contexts. By delineating the relationship between surgical intervention and survival outcomes according to the histologic subtype, we hope to inform future treatment guidelines and improve the prognosis for patients affected by this rare and challenging malignancy.

## 2. Materials and Methods

### 2.1. Patient Characteristics and Variables’ Definition

In this study, we utilized data from the SEER database spanning from 2000 to 2019 to identify a cohort of patients aged 18 years or older with known follow-up information and a defined primary site of origin. We excluded cases with data obtained solely from autopsy reports and those with missing information on the M stage (metastasis status). Eligible patients included those diagnosed with pelvic non-visceral sarcomas, specifically defined by the International Classification of Diseases for Oncology, 10th Edition (ICD-O-10) site code C49.5, as well as those with sarcomas of the bladder (ICD-O site codes C67.0-C67.9 [18,19]) and prostate (ICD-O site code C61.9 [13,20,21]).

To further refine our cohort, we focused on three predominant histologic subtypes, namely liposarcoma (ICD-O histology code 8850/3-8855/3 and 8858/3 [16]), leiomyosarcoma (ICD-O histology code 8890/3), and sarcoma NOS (ICD-O histology code 8800/3 [22]).

We classified tumors as low-grade (grades 1 and 2) or high-grade (grades 3 and 4) based on prior studies that have demonstrated the relevance of these distinctions in predicting outcomes [23,24]. Additional demographic variables such as age, sex, and tumor size were also collected to adjust for potential confounders in the analysis.

### 2.2. Statistical Analyses

To investigate the impact of surgical resection on CSM and other-cause mortality (OCM) across different histologic subtypes and stages, we employed several statistical approaches. Initially, cumulative incidence plots were generated to visually represent the rates of CSM and OCM according to the histologic subtype, stage, and surgical resection status. This visual representation allows for a straightforward comparison of mortality outcomes between different patient groups.

Subsequently, we applied multivariable competing risk regression models to evaluate the independent effect of the histologic subtype on CSM while adjusting for OCM. This approach is particularly important in the context of sarcoma research, as it enables a more accurate assessment of the true CSM risk by accounting for deaths from other causes, which could otherwise bias the results. The Fine and Gray method for competing risk regression was utilized to provide more precise estimates [25]. This method helps mitigate the potential overestimation of OCM by considering the possibility that censoring due to CSM could reduce the number of patients at risk for non-cancer-related mortality.

Further analyses were conducted using separate multivariable competing risk models to explore the role of surgical resection as a predictor of CSM in a histologic subtype- and stage-specific manner. These models were designed to test the hypothesis that surgical resection may confer a survival benefit in both non-metastatic and metastatic settings, but with potentially varying effects based on histology. By stratifying the analyses according to the histologic subtype and stage, we aimed to delineate more precisely the clinical contexts in which surgical intervention may be beneficial.

All statistical tests were two-sided and a significance level of *p* < 0.05 was applied throughout. Analyses were performed using the R Software Environment for Statistical Computing and Graphics (R version 4.1.3, R Foundation for Statistical Computing, Vienna, Austria) [26].

## 3. Results

### 3.1. Patient and Tumor Characteristics

In the study cohort of 2492 soft tissue pelvic sarcoma patients, the distribution of histologic subtypes was as follows: 1017 patients (41%) had liposarcoma, 976 patients (39%) had leiomyosarcoma, and 498 patients (20%) were diagnosed with sarcoma NOS (Table 1).

**Figure 1:**
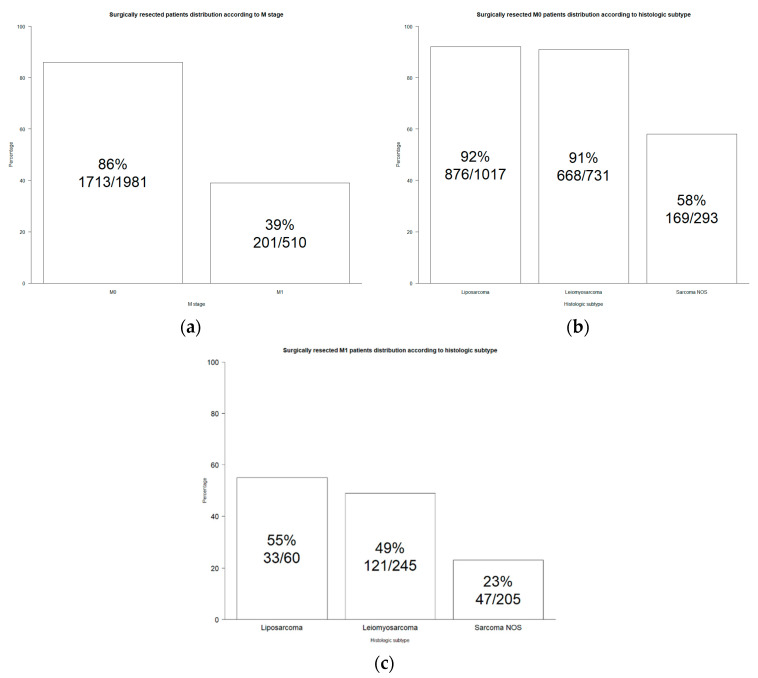
Bar plot of soft tissue pelvic sarcoma diagnosed in 2000–2019 Surveillance, Epidemiology, and End Results database. (**a**) Surgical resection distribution according to M stage; (**b**) surgical resection distribution according to histologic subtype in M0 stage; (**c**) surgical resection distribution according to histologic subtype in M1 stage.

Overall, 86% of non-metastatic and 39% of metastatic patients underwent surgical resection (Figure 1a).

Specifically, surgical intervention was most common in non-metastatic cases of liposarcoma and leiomyosarcoma, with 92% and 91% of patients undergoing resection, respectively. In contrast, only 58% of non-metastatic sarcoma NOS patients underwent surgical resection (Figure 1b). Among metastatic patients, the rates of surgical resection were lower, with 55% in liposarcoma, 49% in leiomyosarcoma, and 23% in sarcoma NOS patients (Figure 1c). The stage at diagnosis differed significantly by subtype, with sarcoma NOS patients more frequently presenting with metastatic disease (41%), followed by leiomyosarcoma (25%) and liposarcoma (6%, *p* < 0.001).

### 3.2. The Effect of Surgical Resection on Cancer-Specific Mortality in Non-Metastatic Patients

**Figure 2:**
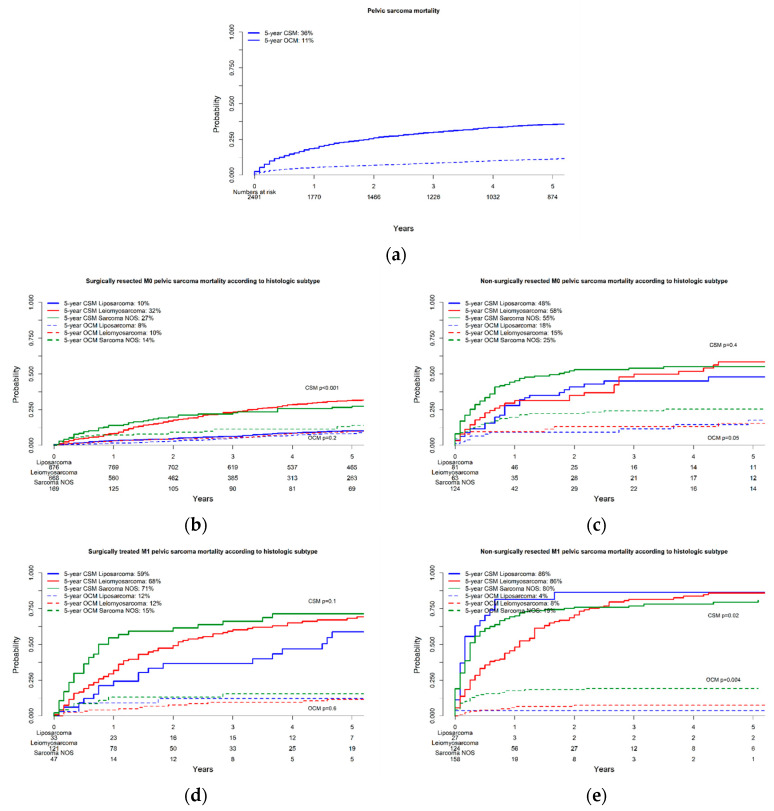
Cumulative incidence plots depicting cancer-specific mortality and other-cause mortality over 10 years in patients with soft tissue pelvic sarcoma diagnosed in 2000–2019 Surveillance, Epidemiology, and End Results database. (**a**) Overall; (**b**) in surgically resected M0 patients according to histologic subtype; (**c**) in non-surgically resected M0 patients according to histologic subtype; (**d**) in surgically resected M1 patients according to histologic subtype; (**e**) in non-surgically resected M1 patients according to histologic subtype.

In patients with non-metastatic soft tissue pelvic sarcoma who underwent surgical resection, the cumulative incidence of CSM at five years varied by histologic subtype. Specifically, liposarcoma patients had the lowest five-year CSM rate at 10%, followed by leiomyosarcoma patients at 32% and sarcoma NOS patients at 27% (Figure 2b).

In non-metastatic patients who did not undergo surgical resection, five-year CSM rates were 48 vs. 58 vs. 55% in liposarcoma vs. leiomyosarcoma vs. sarcoma NOS patients, respectively (Figure 2c). Finally, in separate multivariable competing risk regression models, surgical resection independently predicted lower CSM rates in non-metastatic liposarcoma (HR: 0.2), leiomyosarcoma (HR: 0.5), and sarcoma NOS (HR: 0.4) patients after adjustment for OCM (Table 2).

### 3.3. The Effect of Surgical Resection on Cancer-Specific Mortality in Metastatic Patients

For metastatic soft tissue pelvic sarcoma patients who underwent surgical resection, the five-year CSM rates were considerably higher compared to non-metastatic cases, with 59% for liposarcoma, 68% for leiomyosarcoma, and 71% for sarcoma NOS (Figure 2d). In those who did not undergo surgery, the CSM rates were even higher, with five-year rates of 86% for both liposarcoma and leiomyosarcoma and 80% for sarcoma NOS (Figure 2e). Finally, in separate multivariable competing risk regression models, surgical resection was found to independently predict lower CSM in metastatic leiomyosarcoma patients, with an HR of 0.6 after adjusting for OCM. However, the same was not observed in metastatic sarcoma NOS patients, where the HR was 0.7 (*p* = 0.1), indicating no significant impact of surgery on CSM after adjustment for OCM. Due to the limited number of observations, statistical analyses could not be performed for metastatic liposarcoma cases.

## 4. Discussion

In the current study, we tested for the association between surgical resection status in non-metastatic and metastatic soft tissue pelvic sarcoma, according to the histologic subtype. We addressed the three most frequent histologic subtypes, where sufficient numbers of observations could be identified to justify inclusion in the study, providing meaningful results and conclusions. Several important observations were made.

First, our analyses allowed a large-scale assessment of the three most frequent soft tissue pelvic sarcoma histologic subtypes, according to the non-metastatic vs. metastatic stage. Overall, the most frequent histologic subtype in soft tissue pelvic sarcoma was liposarcoma (41%), followed by leiomyosarcoma (39%), followed by sarcoma NOS (20%), in that order. A comparison of these rates cannot be made with other soft tissue pelvic sarcoma large-scale studies, since no such reports exist [27]. Interestingly, the distribution of more frequent histologic subtypes observed in the present cohort remarkably differed from the retroperitoneal sarcoma cohort analyzed by Nazzani et al. [23] (year of diagnosis: 2004–2014, n = 1226). Specifically, among surgically resected patients, Nazzani et al. identified 68% liposarcoma (47% in the present cohort), 26% leiomyosarcoma (41% in the present cohort), and 6% sarcoma NOS (11% in the present cohort) patients. This comparison suggests a pronounced difference in histologic subtype distribution between soft tissue pelvic sarcoma and retroperitoneal sarcoma. As a consequence, specific studies addressing sarcomas within select sites of origin, such as the current one, are clearly needed [28]. Conversely, extrapolations from studies that are based on different sites of origin should not be made and may be extremely misleading, as evidenced by the above comparison between retroperitoneal sarcoma and pelvic sarcoma.

Moreover, in the present cohort, important differences according to patient and tumor characteristics were also reported. For example, pronounced differences in grade distribution according to histologic subtypes were reported. Specifically, 28% liposarcoma vs. 44% leiomyosarcoma vs. 59% sarcoma NOS patients harbored high-grade tumors. Last but not least, we recorded sex differences according to histologic subtype. However, sex did not represent an independent predictor of CSM in any of the models fitted within the current study. These differences justify and require the use of multivariable adjustment to avoid the effects of bias and confounding. Similarly, we recorded a non-negligible risk of OCM (11% in the overall cohort). As a consequence, the use of competing risk regressions is recommended, since reporting CSM without adjustment for OCM may also result in bias and confounding. These statistical methods were used in the current study but not in other contemporary studies, since such studies do not exist.

Second, regarding surgical resection status, virtually all non-metastatic pelvic liposarcoma (92%) and leiomyosarcoma (91%) patients benefited from surgical resection vs. only a slight majority in sarcoma NOS patients (58%). A similar rank order of lesser absolute value was recorded for surgical resection status in metastatic soft tissue pelvic sarcoma. Specifically, 55% of liposarcoma, 49% of leiomyosarcoma, and 23% of sarcoma NOS patients benefited from surgical resection. These observations validate the central role of surgical resection status in the vast majority of non-metastatic pelvic liposarcoma (92%) and leiomyosarcoma (91%), as well as in most pelvic sarcoma NOS patients (58%).

Interestingly, elevated rates of surgical resection were also recorded in metastatic soft tissue pelvic sarcoma, where the majority of pelvic liposarcoma (55%), virtually half of the leiomyosarcoma (49%), and as many as 23% of sarcoma NOS patients underwent surgical resection. These observations also attest to the confidence in surgical resection even in metastatic soft tissue pelvic sarcoma, predominantly when its histologic subtype does not include sarcoma NOS. Taken together, surgical resection has a pivotal role in the management of pelvic soft tissue sarcoma, which even applies to metastatic patients.

Third, in non-metastatic soft tissue pelvic sarcoma, CSM rates of surgically resected patients were invariably better than those of non-surgically resected patients. The association between surgical resection and lower CSM, even after the most complete multivariable adjustment, was clearly and convincingly strongest in liposarcoma (HR: 0.2), followed by sarcoma NOS (HR: 0.4), followed by leiomyosarcoma patients (HR: 0.6). These observations validate, albeit in a retrospective fashion where selection bias may still be operational, a significantly lower CSM when surgical resection was applicable in soft tissue pelvic sarcoma. Variations in resection benefits according to histology may be explained by different biological behaviors. Specifically, patients affected by histotypes at high risk of local recurrence (such as liposarcoma) could experience a stronger CSM benefit after radical curative surgery. Conversely, in sarcomas at high risk of distant recurrence (such as leiomyosarcoma), radical surgery less frequently represents the definitive treatment, especially in locally advanced conditions [2,5].

Fourth, in metastatic soft tissue pelvic sarcoma, regarding surgical resection status, it is highly noteworthy that an absolute and relative benefit was recorded in leiomyosarcoma (HR: 0.6). Conversely, no association between CSM and surgical resection could be identified in multivariable competing risk analyses for sarcoma NOS (HR: 0.7, *p* = 0.1). Finally, the effect of surgical resection could not be examined in metastatic pelvic liposarcoma due to the rarity of these patients. As a consequence, surgical resection should be strongly considered in metastatic pelvic leiomyosarcoma; conversely, it should be carefully considered in metastatic pelvic sarcoma NOS.

The observations made in the current study can be summarized. The present study represents the only large-scale contemporary analysis of soft tissue pelvic sarcoma patients [27]. It illustrates that three histologic subtypes account for the majority and that only these three histologic subtypes could be validly examined and interpreted. Important patient and tumor heterogeneity exist within these three soft tissue pelvic sarcoma patient groups. As such, multivariable analyses are required. Additionally, a non-negligible proportion of patients (11%) died of other causes at five years. In consequence, a competing risk regression is required to avoid the effect of bias or confounding due to OCM. In non-metastatic soft tissue pelvic sarcoma, surgical resection applies to virtually all liposarcoma and leiomyosarcoma patients and most sarcoma NOS patients. In metastatic soft tissue pelvic sarcoma, the majority of pelvic liposarcoma, virtually half of leiomyosarcoma, and as many as 23% of sarcoma NOS patients underwent surgical resection. In non-metastatic patients, the most important association between surgical resection and lower CSM in absolute and relative terms applies to liposarcoma, followed by sarcoma NOS, followed by leiomyosarcoma patients. In metastatic patients, surgical resection is associated with lower CSM only in leiomyosarcoma patients.

Despite the novel insights provided by our study, several limitations need to be addressed. Firstly, the retrospective nature of the SEER database may introduce inherent selection biases, as data are collected from various institutions with different patient management protocols. This variability can potentially skew the findings and limit the generalizability of our results. However, databases like SEER and the National Cancer Database remain indispensable for investigating rare primary malignancies, such as soft tissue pelvic sarcomas, as they allow for the accumulation of large patient cohorts necessary for a robust statistical analysis and to draw meaningful conclusions. Secondly, the SEER database lacks crucial clinical details, including local recurrence rates, metastatic progression, preoperative and postoperative treatment modalities, and predictors of cancer control outcomes, such as resection margin status [29,30,31]. These factors are known to significantly influence survival outcomes in sarcoma patients. The absence of these variables limits the ability to perform a comprehensive analysis of factors impacting CSM. For example, stratification according to margin status could probably enhance the protective role of radical surgery in pelvic soft tissue sarcoma. In fact, due to the intricate anatomy and limited space within the pelvis, achieving microscopically clear margins in pelvic soft tissue sarcoma can be quite difficult, often necessitating extensive and high-risk surgical resections. Despite this consideration, surgical resection still represents a strong predictor of lower CSM as for other anatomic sites, especially in subtypes characterized by higher local recurrence rates. Moreover, risk stratification according to microscopic vs. macroscopic margin involvement and the histologic subtype would represent a highly relevant aspect in preoperative clinical decision-making. Thirdly, the SEER database does not include specific comorbidity data, which precludes adjusting for underlying health conditions that might affect survival outcomes independently of sarcoma treatment. While we attempted to mitigate this limitation by incorporating OCM rates into our analysis, this approach is not a substitute for detailed comorbidity data. Understanding the interplay between patient health status and treatment efficacy is crucial, especially in elderly populations or those with multiple comorbid conditions. Moreover, while we identified several (sixteen) additional histologic subtypes of soft tissue pelvic sarcoma in the database, such as solitary fibrous tumors (55 patients), the sample sizes for these rarer subtypes were too small to include in meaningful statistical analyses. This limited our ability to generalize findings to all pelvic sarcoma subtypes and highlights the need for larger, multicentric studies to validate our observations across diverse histologic categories. In conclusion, while our study offers valuable insights, these limitations underscore the necessity for future research to incorporate more comprehensive clinical data and broader patient populations to fully elucidate the impact of surgical resection on survival outcomes in soft tissue pelvic sarcoma.

## 5. Conclusions

In cases of non-metastatic soft tissue pelvic sarcoma, surgical resection could be associated with reduced CSM. However, this protective association could apply specifically to leiomyosarcoma in metastatic patients.

## Figures and Tables

**Table 1:** Descriptive characteristics of patients diagnosed with soft tissue pelvic sarcoma between 2000 and 2019 in the Surveillance, Epidemiology, and End Results database according to most frequent histologic subtypes. Data are shown as medians for continuous variables or as counts and percentages (%) for categorical variables. IQR: interquartile range.

Pelvic Soft Tissue Sarcoma	Overall n = 2491	Liposarcoma n = 1017 (41%)	Leiomyosarcoma n = 976 (39%)	Sarcoma NOS n = 498 (20%)	*p*-Value
**Age at diagnosis (years)**					
Median (IQR)	62 (52–72)	62 (51–71)	62 (51–71)	65 (52–75)	0.002
**Sex—**male	1358 (55%)	731 (72%)	365 (37%)	262 (53%)	<0.001
**Surgical resection**	1914 (77%)	909 (89%)	789 (81%)	216 (43%)	<0.001
**Grade**					
High-grade	1013 (41%)	286 (28%)	434 (44%)	293 (59%)	<0.001
Unknown	566 (23%)	129 (13%)	284 (29%)	153 (31%)	
**Tumor size (mm)**					
Median (IQR)	100 (60–150)	115 (73–170)	88 (50–130)	105 (70–146)	<0.001
**M stage**					
M1	510 (20%)	60 (6%)	245 (25%)	205 (41%)	<0.001
**Tumor origin**					
Non-visceral	2303 (92%)	1017 (100%)	843 (86%)	443 (89%)	<0.001
Visceral	188 (8%)	0 (0%)	133 (14%)	55 (11%)	
Bladder	131 (5%)	0 (0%)	91 (9%)	40 (8%)	
Prostate	57 (2%)	0 (0%)	42 (4%)	15 (3%)	

**Table 2:** Separate competing risk regression models predicting cancer-specific mortality after adjustment for other-cause mortality according to each histologic subtype. All patients were diagnosed with soft tissue pelvic sarcoma between 2000 and 2019 in the Surveillance, Epidemiology, and End Results database.

	Non-Metastatic				Metastatic			
	Hazard Ratio	95% CI	*p*-Value	Non-Resected/Resected	Hazard Ratio	95% CI	*p*-Value	Non-Resected/Resected
**Liposarcoma**								
Surgical resection status (no vs. yes)	0.2	(0.1–0.3)	<0.001	171/876	-	-	-	27/33
**Leiomyosarcoma**								
Surgical resection status (no vs. yes)	0.5	(0.3–0.8)	<0.001	63/668	0.6	(0.4–0.8)	<0.001	121/124
**Sarcoma NOS**								
Surgical resection status (no vs. yes)	0.4	(0.2–0.6)	<0.001	169/124	0.7	(0.5–1.1)	0.1	47/158

## Data Availability

Data available in a publicly accessible repository.
